# Mesothelioma presenting as a breast lump: when is a breast lump not a breast lesion?

**DOI:** 10.1259/bjrcr.20150003

**Published:** 2015-07-17

**Authors:** S L Savaridas, G D Bristow

**Affiliations:** ^1^ North Tyneside General Hospital, North Sheilds, UK; ^2^ Radiology Department, North Tyneside General Hospital, North Sheilds, UK

## Abstract

A case of mesothelioma presenting as a breast lump. Relevant imaging is reviewed and the differential diagnoses discussed. The absence of the pectoralis muscle on mammogram is a potentially ominous sign which should not be assumed to be due to technical factors.

## Summary

A case of mesothelioma presenting as a breast lump. The absence of the pectoralis muscle on mammogram is a potentially ominous sign that should not be assumed to be due to technical factors.

## Clinical presentation

A 71-year-old female presented to the symptomatic breast clinic with a palpable mass in the left upper outer quadrant (LUQ) of the breast. As per the National Institute for Clinical Excellence guidelines,^1^ all patients presenting to the one-stop symptomatic breast clinic receive a triple assessment with clinical assessment, imaging (mammography and focused breast ultrasound) and biopsy.

Clinical examination identified a suspicious thickening that felt fixed to the pectoralis muscle in the LUQ. There were no associated skin or nipple changes. This was considered likely malignant and graded E4/5.

**Figure 1.**
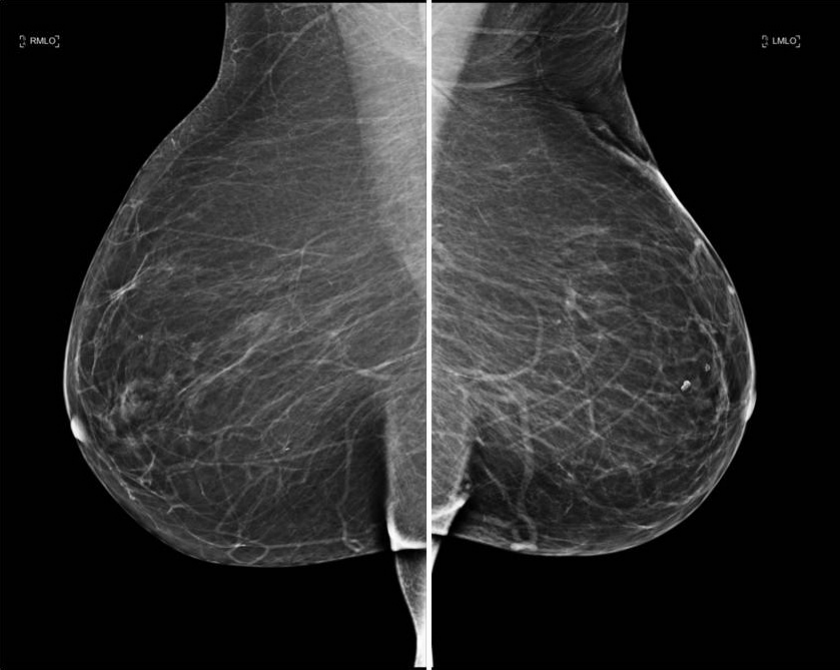
Mediolateral oblique mammographic view demonstrating ominous absence of full pectoralis muscle shadow on the left.

**Figure 2.**
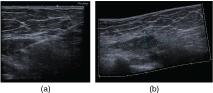
(a) Ultrasound demonstrating a mass deep within the breast tissue. (b) Ultrasound-guided biopsy image demonstrating biopsy tract.

Initial mammography (Figure 1) was essentially normal and reported as M1 (no abnormality). She subsequently had an ultrasound scan (Figure 2) of the symptomatic breast. There was no abnormality identified at the symptomatic site (LUQ). However, deep in the posterior medial breast was an ill-defined hypoechoic expansion measuring 6 × 2 × 4 cm that seemed to be arising from the pectoralis muscle. This was discussed with the surgeons who felt that the appearance corresponded to the abnormality they could palpate. Three 14-gauge core needle biopsies were taken and the needle tract documented.

## Differential diagnosis

The initial differential diagnosis was broad and included breast cancer, sarcoma, bruising and lipoma.

## Investigations/imaging findings

**Figure 3.**
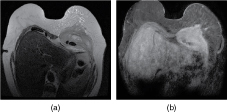
Breast MRI *T*_2_ (a) and contrast-enhanced, fat-saturated *T*_1_ (b) of mesothelioma.

Further imaging was performed; a breast MRI (Figure 3) was arranged 3 working days later. A large (16 × 12 × 7 cm) mass was seen arising from and replacing most of the pectoralis major muscle in the left breast. It engulfed the lower costochondral area and extended posteriorly into the thoracic cavity, involving a large portion of the anterior chest wall. The breast tissues anterior to the pectoralis mass were oedematous but there was no identifiable discrete breast lesion. In addition, there was a left-sided moderate-sized pleural effusion compressing the underlying lung. Sarcoma involving the anterior chest wall was considered the most likely differential diagnosis.

**Figure 4.**
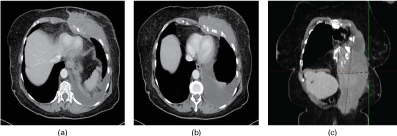
CT axial (a,b) and coronal (c) views demonstrating large soft-tissue mass extending into rectus abominus, with associated rib destruction, pleural effusions and restriction of the left hemithorax.

Subsequent CT staging scan (Figure 4) demonstrated a large soft-tissue mass involving the muscles of the left lateral chest wall and extending inferiorly to involve and expand the left rectus abdominis. Involvement of the costal cartilages and pleura was seen with lobulated pleural thickening and a large left pleural effusion. There was further soft tissue in the left superior mediastinum with neurovascular encasement of the left subclavian artery, vein and brachial plexus. The differential diagnosis remained wide, including a soft-tissue sarcoma, lymphoma, left pancost tumour and desmoid tumour of the rectus abdominis muscle. Histopathological results from the original core biopsy diagnosed mesothelioma.

## Treatment and follow-up

The patient was discussed with the breast and lung multidisciplinary teams before being referred to oncology for consideration of palliative chemo-radiotherapy.

## Discussion and learning points

The majority of palpable breast lumps are benign, the most common being a fibroadenoma. Of those lesions that are malignant, most are invasive ductal carcinomas. As with our patient, malignant masses tend to be hard, immobile and fixed to the surrounding skin and soft tissues, with poorly defined margins.^2^

To our knowledge, there is only one other case in the literature, published 9 years ago, of a mesothelioma presenting as a breast lump.^3^ Our case demonstrates much more extensive disease. Also, most likely, owing to changes in imaging practices, our case is the only case in the published literature with breast MR appearances of mesothelioma. Interestingly, the patient in the previous case had a screening mammogram reported as normal 2 months prior to presentation with a breast lump. On the mediolateral oblique (MLO) image included in their article, the pectoralis muscle cannot be seen. The normal contralateral MLO image is not included for comparison. Guidelines state that, on MLO views, the pectoral muscle shadow should be visible at nipple level.^4^ While the absence of a pectoralis muscle is frequently due to technical difficulties, in both ours and the previous case, it appears that the absence was almost certainly due to the underlying disease process and was, therefore, highly significant.

Mesothelioma is a fatal cancer with a dismal prognosis. Mortality in the UK is expected to peak at 2450 deaths per year by 2015. A review of 146 patients diagnosed with mesothelioma over a 4-year period in Leeds calculated a median survival from time of diagnosis of just 267 days (approximately 8.9 months). In their series, only 35% of patients were considered fit for palliative chemotherapy, and of those just over half declined.^5^

The majority of the patients who develop mesothelioma have documented evidence of definite or probable asbestos exposure.^5^ Although our patient had not been aware of asbestos exposure prior to diagnosis, on close questioning, she admitted washing the clothes of her husband who worked as joiner. It is recognized that women are more likely to gain asbestos exposure domestically.^6^

There are case reports of seeding along biopsy tract in mesothelioma.^7^ However, only 7 (5%) of the 146 patients in the population-based review developed tumour invasion of the biopsy tract, although almost all of these patients had had prophylactic radiotherapy to these sites.^5^ This suggests that there is no need for undue concern if a mesothelioma is diagnosed unexpectedly following a core biopsy as in our case.

## Conclusions

This case report emphasizes that the absence of the pectoralis muscle on mammogram is a potentially ominous sign that should not be assumed to be due to technical factors. Not all “breast lumps” arise from the breast tissue and it is vitally important that tissue down to chest wall is fully imaged, both on ultrasound and mammogram.
